# Production and Characterization of Nanoparticulate Polyelectrolyte Complexes of Chitosan–Catechol, Ulvan, and Hyaluronic Acid

**DOI:** 10.1021/acsomega.4c07429

**Published:** 2025-01-17

**Authors:** Francisco J. Caro-León, Erika Silva-Campa, René A. Navarro-López, Daniel Fernández-Quiroz, Vivian A. Figueroa-León, Maria A. Trujillo-Ramirez, Luis Miguel López-Martínez, Maria Rosa Aguilar, Osiris Álvarez-Bajo

**Affiliations:** † Instituto de Ciencia y Tecnología de Polímeros (ICTP) CSIC, 28006 Madrid, Spain; ‡ Departamento de Investigación en Física de la Universidad de Sonora (DIFUS), 83000 Hermosillo Sonora, Mexico; § Departamento de Ingeniería Química y Metalurgia, Universidad de Sonora, 83000 Hermosillo Sonora, Mexico; ∥ Programa de Ingeniería Biomédica, Universidad de Sonora, 83000 Hermosillo Sonora, Mexico; ⊥ Departamento de Investigación en Polímeros y Materiales (DIPM), Universidad de Sonora, 83000 Hermosillo Sonora, Mexico; # Universidad Estatal de Sonora (UES), Av. Ley Federal del Trabajo s/n, Col. Apolo, 83100 Hermosillo, Sonora, Mexico; ¶ CIBER de Bioingeniería, Biomateriales y Nanomedicina, Instituto de Salud Carlos III, 28029 Madrid, Spain; ∇ Consejo Nacional de Humanidades Ciencia y Tecnología CONAHCyT, Ave. Insurgentes Sur 1582, Col. Crédito Constructor, Benito Juárez 03940, CDMX, Mexico

## Abstract

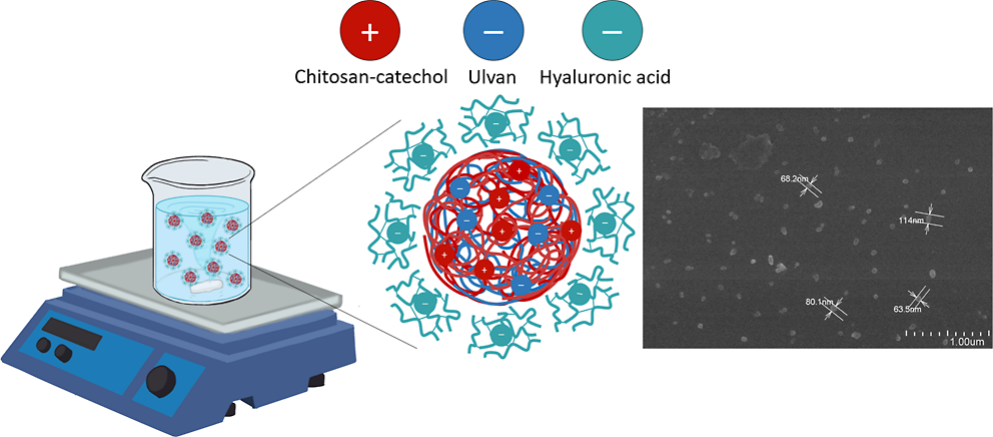

This work provides the first description of the synthesis and characterization of polymeric nanoparticles (NPs) obtained by polyelectrolyte complexes (PECs) of three polysaccharides: chitosan (Cs) or its catecholic derivative, ulvan, and hyaluronic acid (HA). The carbodiimide method used catechol group conjugation onto the Cs backbone using the catechol-containing compound hydrocaffeic acid. A degree of substitution of 2.98% was determined by spectroscopy in the chitosan–catechol (CsC) conjugate. DLS studies showed hydrodynamic diameter (*D*_h_) close to 300 nm for PECs of Cs and CsC with ulvan, with positive zeta potential for both complexes. HA coating was confirmed by obtaining negative zeta potential. The HA coating reduced the *D*_h_ values of the materials by approximately 100 nm. This resulted in maintaining stability in water suspension for up to 8 weeks, yielding unimodal size distribution. ATR-FTIR spectra confirmed the presence of the polyelectrolytes in the PECs. FE-SEM studies demonstrated that HA-coated NPs exhibited smaller sizes than their uncoated counterparts. PECs with CsC maintained higher free radical scavenging activity than those with unmodified Cs, with the HA coating only reducing antioxidant activity by 16%. All tested NPs demonstrated biocompatibility at concentrations up to 200 μg/mL in ARPE-19 cell cultures derived from human retinal pigment epithelial cells.

## Introduction

Polyelectrolytes (PEs) are macromolecules characterized by repeat units that dissociate into highly charged polymers when placed in an ionizing solvent, forming either positively or negatively charged chains.^1^ These PEs can interact with oppositely charged ions to create polyelectrolyte complexes (PECs). PECs arise primarily through cooperative electrostatic interactions between polycations and polyanions when their aqueous solutions are mixed. This leads to a dense phase separate from the solvent without requiring chemical cross-linkers.^2^ The formation of PECs typically occurs in three stages: initially, primary complexes form through Coulomb forces; second, intracomplexes undergo a formation process that may involve new bond formation or adjustment of polymer chain distortions; finally, intercomplex aggregation occurs, primarily through hydrophobic interactions.^3^ PECs are insoluble in most common solvents, and their elasticity varies with moisture content—appearing rubbery under wet conditions and hard and brittle when dry. They are permeable to all electrolytes but block macroparticles.^4^ In recent years, PECs have gained significant interest due to their versatile applications, including uses in membranes, film coatings, protein isolation, targeted nucleic acid delivery, pharmaceutical binding, microcapsule preparation for drug delivery, dialysis membranes, contact lenses, enzyme mimics, medical uses, and nanoparticles (NPs) for targeted tissue delivery.^4−8^

Chitosan (Cs), a linear cationic biopolymer, is produced by deacetylation of chitin, which ranks as the second most prevalent biopolymer in nature after cellulose. Chitin is a key structural element found in diverse eukaryotic organisms such as crustaceans, insects, and fungi.^9,10^ Chemically, Cs is a β-(1 → 4)-linked linear copolymer of d-glucosamine and *N*-acetyl-d-glucosamine,^11^ with its polymeric structure featuring numerous amino and hydroxyl functional groups. This versatility allows for various modifications, facilitating derivatization and paving the way for exploration across a wide array of applications.^12−14^ Among the numerous existing Cs derivatives, those containing the catechol moiety stand out significantly. This moiety is naturally found as a side chain of l-3,4-dihydroxyphenylalanine, an amino acid crucial for the strong underwater adhesion of mussels. Chitosan–catechol (CsC) has shown not only excellent solubility in neutral pH solutions but also strong adhesiveness to tissue surfaces and enhanced antioxidant activity.^15^

Because of the protonation of amino groups along its backbone, Cs transitions into a cationic PE in acidic environments. This property enables it to form PECs with negatively charged PEs. Numerous categories of polyanions have been thoroughly explored for their compatibility with Cs, including various natural polymers such as alginate, pectin, carrageenan, carboxymethyl cellulose, gelatin, ulvan, and hyaluronic acid,^16−22^ and synthetic polymers.^23,24^ Based on different preparation methods, the Cs-based PECs can be developed to form different materials, including hydrogels,^25−28^ films,^29,30^ microparticles,^31^ and NPs.^32−35^ Cs-based PECs have been proven to possess various applications in biomedical and pharmaceutical areas, such as drug delivery and controlled release.^36,37^

Green macroalgae species belonging to the genus *Ulva* (Chlorophyta) are recognized as edible seaweeds containing a spectrum of health-promoting bioactive compounds. *Ulva* is particularly abundant in dietary fiber, which not only promotes gastrointestinal health but also activates the body’s own antioxidant system. This helps safeguard the body from harmful oxidation products that can lead to diseases including chronic inflammation and cancer. Among the essential components of *Ulva* is the soluble fiber ulvan, a sulfated polysaccharide with significant biological activities. Ulvan exhibits immunomodulatory, antiviral, antioxidant, antihyperlipidemic, and anticancer properties, and it possesses the ability to modulate cellular signaling processes in both plant and animal systems.^38−41^ Ulvan is a polysaccharide found in the cell wall of *Ulva* algae, constituting approximately 9 to 36% of the dry weight of *Ulva* biomass. Its composition predominantly comprises sulfated rhamnose, along with uronic acids such as glucuronic acid and iduronic acid, as well as xylose.^42^

Hyaluronic acid (HA), a major glycosaminoglycan of the extracellular matrix, is a polysaccharide consisting of repetitive polymeric disaccharides of d-glucuronic acid and *N*-acetyl-d-glucosamine. These units are joined by alternating β-(1 → 4) and β-(1 → 3) glycosidic bonds.^43^ HA plays a crucial role in providing lubrication and imparting viscoelastic properties to synovial fluid. By doing so, it effectively reduces friction between joint surfaces, thereby enhancing joint mobility.^44^ Moreover, HA can modulate inflammation by inhibiting leukocyte migration, decreasing the expression of proinflammatory cytokines and enzymes, and suppressing immune responses.^45^ Research has shown that the properties of HA largely depend on its molecular weight. High-molecular-weight HA exhibits anti-inflammatory and immunosuppressive characteristics, while low-molecular-weight HA acts as a strong proinflammatory agent.^46^ HA contributes to tissue repair and remodeling processes by promoting angiogenesis, extracellular matrix remodeling, and cell migration.^47^ Additionally, HA is frequently cited in the literature as a component of theranostic agents for cancer owing to its strong receptor binding to CD44, which is overexpressed in various solid tumors, enabling differentiation between healthy and malignant tissues. This binding affinity has been instrumental in the development of HA-based nanocarriers, which exhibit preferential tumor accumulation and enhanced cellular uptake.^48,49^

This study presents a comprehensive investigation into the synthesis and characterization of nanoparticulate PECs derived from Cs or its catecholic derivative, ulvan, and HA. To the best of our knowledge, no material obtained through electrostatic interactions between these three polysaccharides has been reported in the existing literature. Considering the polyelectrolytic nature of Cs and its derivatives, as well as ulvan and HA, the objective of this work is to produce nanoparticulate PECs with high colloidal stability in aqueous suspension, combining all of the bioactive properties of their components, such as mucoadhesiveness, antimicrobial capacity, antioxidant activity, and cell surface receptor binding. This broadens the potential applications of these PECs while retaining their biocompatibility. Future research endeavors may explore the capacity of these NPs to integrate molecules with specific biological activities into their structures and subsequently release them at targeted sites.

## Experimental Section

### Materials

Cs 90/200 was purchased from Chitoscience (Halle, Germany) with a viscosity of 151–350 mPas (1% in 1% acetic acid, 20 °C) and deacetylation degree of 87.6–92.5% provided by the supplier. Ulvan-*Ulva armoricana*-winter-heavy was supplied by Cymit Quimica S.L (Barcelona, Spain). HA with a molecular weight of 200–300 kDa was provided by Bioiberica (Barcelona, Spain). 3,4-Dihydroxyhydrocinnamic acid, 98% (hydrocaffeic acid, HCA), *N*-(3-(dimethylamino)propyl)-*N′*-ethylcarbodiimide hydrochloride (EDC), 2,2-diphenyl-1-picrylhydrazyl (DPPH), DCl, D_2_O (99.9 atom % D), CH_3_COOH, HCl, and NaOH were purchased from Merck KGaA (Darmstadt, Germany) and utilized without additional purification.

### Synthesis of CsC Derivative

**Scheme 1:**
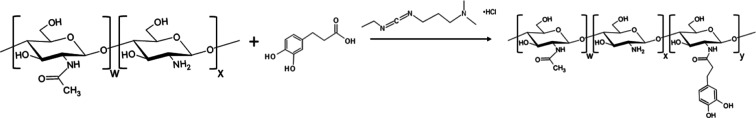
Synthetic Procedure of CsC

The CsC conjugate was synthesized at room temperature using standard EDC chemistry, following a previously reported method, as illustrated in Scheme 1.^50^ Briefly, in a total volume of 5.4 mL of 1 N HCl, 62 mg (equivalent to 0.385 mmol monosaccharide residue) of Cs was dissolved under magnetic stirring. Following this, the pH of the polysaccharide solution was adjusted to 5.5 using 0.2 N NaOH. Subsequently, a solution containing 7.5 mg (0.041 mmol) of HCA in 0.32 mL of deionized water (DW) was added. Then, 18.3 mg (0.118 mmol) of EDC dissolved in a 5.34 mL mixture of DW and ethanol (1:1 v/v) was slowly added dropwise. The reaction mixture was stirred for 3 h while keeping the pH at 5.5. Following completion of the reaction, the product was dialyzed against DW for 48 h and subsequently recovered by freeze-drying. Throughout the entire reaction process, precautions were taken to minimize phenol oxidation by minimizing exposure to light. To obtain the ^1^H nuclear magnetic resonance (^1^H NMR) spectra, solutions of 5 mg of Cs and CsC in 1 mL of D_2_O/DCl mix (50:1 v/v) were analyzed at 70 °C in a Varian Mercury equipment working at 400 MHz (Palo Alto, USA). The degree of substitution [DS(%)] of catechol was determined in a 2 mg/mL solution of the polysaccharide in 0.1% v/v CH_3_COOH. This was achieved by employing a calibration curve of absorbances obtained from solutions of HCA, measured at a wavelength of 280 nm using a Multiskan GO microplate spectrophotometer (Thermo Fisher Scientific, Waltham, USA). The calibration curve exhibited linearity within the concentration range of 0.009–0.3 mg/mL (*y* = 3.9213*x* + 0.0158, *R*^2^ = 0.999) (see Figure S1). Measurements were conducted in triplicate.

### Preparation and Characterization of Nanoparticulate PECs

**Scheme 2:**
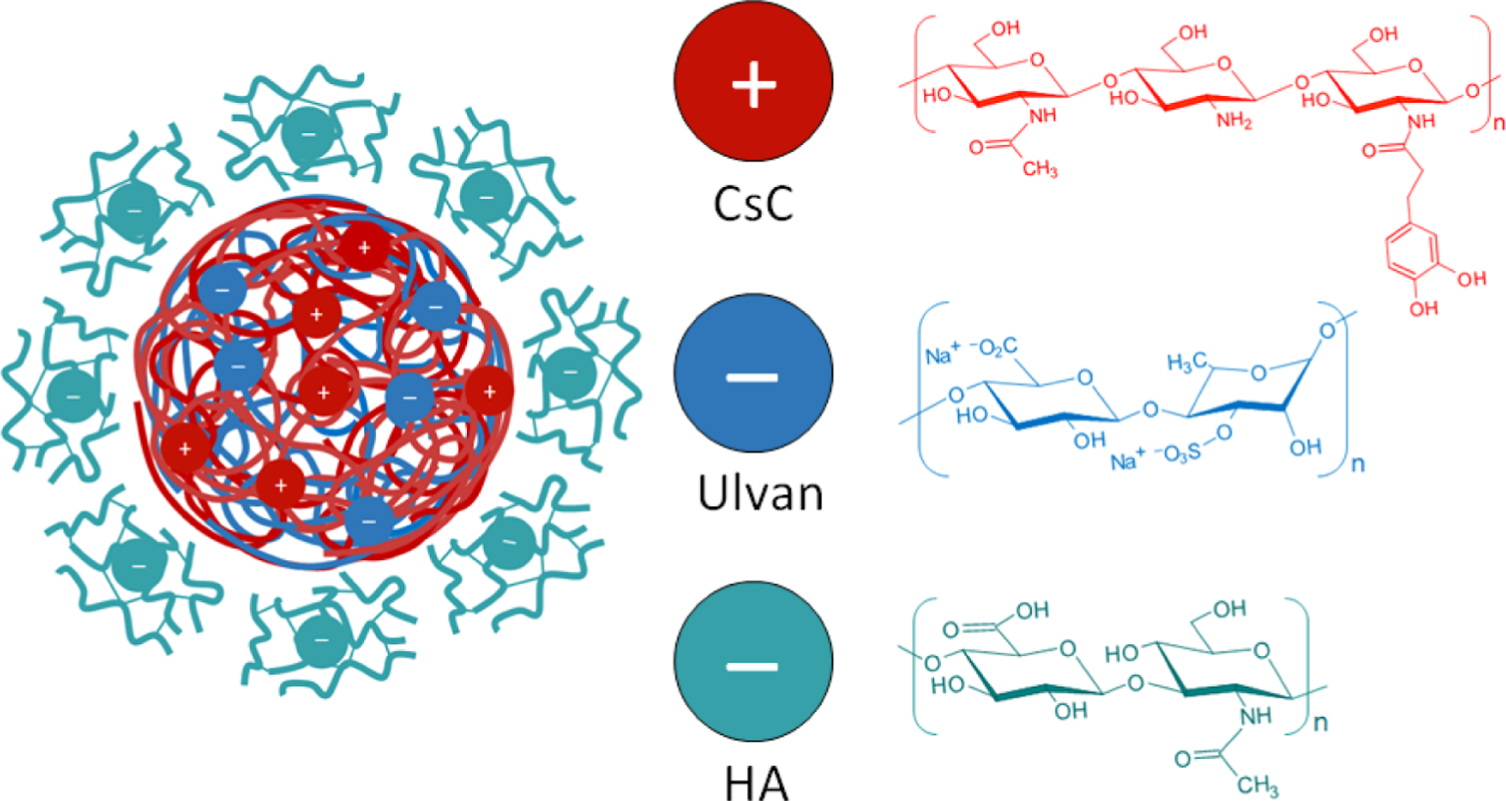
Representation of the PECs

Polymeric PECs were formed using the polyelectrolytic complexation method, which involves the interaction of oppositely charged PEs, as illustrated in Scheme 2. Initially, Cs and CsC were individually dissolved completely in 0.1% (v/v) CH_3_COOH at a concentration of 0.75 mg/mL. The formation of NPs of Cs and CsC (Cs–U NPs and CsC–U NPs, respectively) occurred spontaneously in water suspension when an aqueous solution of ulvan (0.5 mg/mL) was added dropwise into 2 mL of the respective Cs or CsC solution. To use mass ratios from 0.45 to 1.25 of Cs or CsC: ulvan, the initial volume of the chitosans (Cs or CsC) was kept constant, while the volume of ulvan added was varied. This process was conducted under magnetic stirring (950 rpm) at room temperature for 10 min. To enhance colloidal stability, HA-coated NPs were generated by gradually adding different volumes of Cs–U NPs (Cs–U/HA NPs) or CsC–U NPs (CsC–U/HA NPs) to 1 mL of HA aqueous solution (0.5 mg/mL) at 600 rpm, corresponding to NPs/HA volume ratios from 0.15 to 1.15. The coated PECs were stirred for 10 min at room temperature. All samples were prepared in triplicate and immediately subjected to further analysis.

Hydrodynamic diameter (*D*_h_) and polydispersity index (PDI) of the PECs were determined by dynamic light scattering (DLS) using an Anton Paar Litesizer 500 instrument (Graz, Austria). The stability of the NPs was evaluated in terms of the variation of *D*_h_ and PDI. The materials were stored at 4 ± 2 °C during the analysis time (8 weeks). The zeta potential (ζ-potential) of the NPs was measured by laser Doppler electrophoresis using the Litesizer 500. The morphology of the NPs was observed by field emission-scanning electron microscopy (FE-SEM) with a Hitachi SU8000 TED (Tokyo, Japan) cold-emission FE-SEM microscope, operating with an accelerating voltage between 15 and 25 kV. The samples were individually deposited onto copper grids coated with a Formvar membrane, allowed to settle, dried, and subsequently coated with a thin layer of Au/Pd (60:40).

Cs–U NPs, CsC–U NPs, Cs–U/HA NPs, and CsC–U/HA NPs were centrifuged at 5000 rpm for 30 min, using a Heraeus Megafuge 16R centrifuge (Thermo Fisher Scientific, Waltham, USA), and the supernatant was subsequently discarded. The materials were then freeze-dried using a Labconco FreeZone 2.5L freeze-drier (Labconco Corp., Kansas City, USA). After freeze-drying, they were characterized using attenuated total reflection-Fourier transform infrared (ATR-FTIR) spectroscopy. The spectra of these dried PECs were recorded in the mid-infrared absorbance range (4000–500 cm^–1^) using a Nicolet iS 50 FTIR spectrometer (Thermo Fisher Scientific, Waltham, USA).

### Antioxidant Activity of the NPs In Vitro

The radical scavenging activity (RSA) of the NPs was evaluated by the DPPH methodology. Separately, freeze-dried Cs–U NPs, CsC–U NPs, Cs–U/HA NPs, and CsC–U/HA NPs were suspended in 0.1% (v/v) CH_3_COOH under magnetic stirring, at equivalent concentrations of Cs or CsC from 0.5 to 3.0 mg/mL. A volume of alcoholic solution of DPPH (0.125 mM) was added to each NP suspension to generate a 1:1 v/v mixture, which was maintained at constant magnetic stirring for 30 min. Subsequently, the mixtures were centrifuged at 5000 rpm at room temperature for 30 min. The reduction in absorbance relative to the DPPH solution was determined in the supernatant of the suspensions using the microplate reader. The RSA was calculated using the following equation
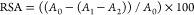
whre, *A*_0_ represents the absorbance of the blank, *A*_1_ represents the absorbance of the sample, and *A*_2_ represents the absorbance of the sample under the same conditions as *A*_1_ but with ethanol replacing the DPPH solution. The results are presented as mean ± standard deviation (*n* = 5).

### Cell Culture and Cytotoxicity Determination of the PECs

ARPE-19 cells from the retinal pigment epithelium were used to determine the cytotoxicity of the PECs because it is a nontumor cell line, which makes it suitable for evaluating the biocompatibility of the materials. Cells were cultured in Dulbecco’s modified Eagle’s medium (DMEM) supplemented with fetal bovine serum (10%), l-glutamine (1%), and penicillin–streptomycin (1%) at 37 °C and 5% CO_2_, on a 96-well flat bottom plate (100 μL/well at a density of 5 × 10^4^ cells/ml). After culturing for 48 h, the cells were washed and incubated with 100 μL of Cs–U NPs, CsC–U NPs, Cs–U/HA NPs, or CsC–U/HA NPs in fresh DMEM (50–200 μg/mL) for 24 h; in the positive control group, the procedure was identical, except the cells were cultured solely with DMEM medium. Each sample was evaluated in triplicate. Subsequently, all wells were washed, and 100 μL of 3-(4,5-dimethylthiazol-2-yl)-2,5-diphenyl-tetrazolium bromide (MTT) dye (0.5 mg/mL) was added to each well, followed by incubation for an additional 4 h at 37 °C. After incubation, the supernatant was discarded, and the formazan crystals were dissolved in 100 μL of DMSO, followed by shaking for 5 min. Absorbance at 570 nm was measured using a microplate reader. The percentage of cell viability [CV(%)] was expressed relative to the positive control. The cell group with DMSO (10% v/v) was taken as the negative control.

### Statistical Analysis

Whenever applicable, the Kruskal–Wallis test was conducted at a 95% confidence level. When significant differences were detected, a median mood test was applied. Data analysis was performed using R version 4.1.2 (www.R-project.org) within the nonparametric package.^51^

## Results and Discussion

### Synthesis of CsC Derivative

**Figure 1:**
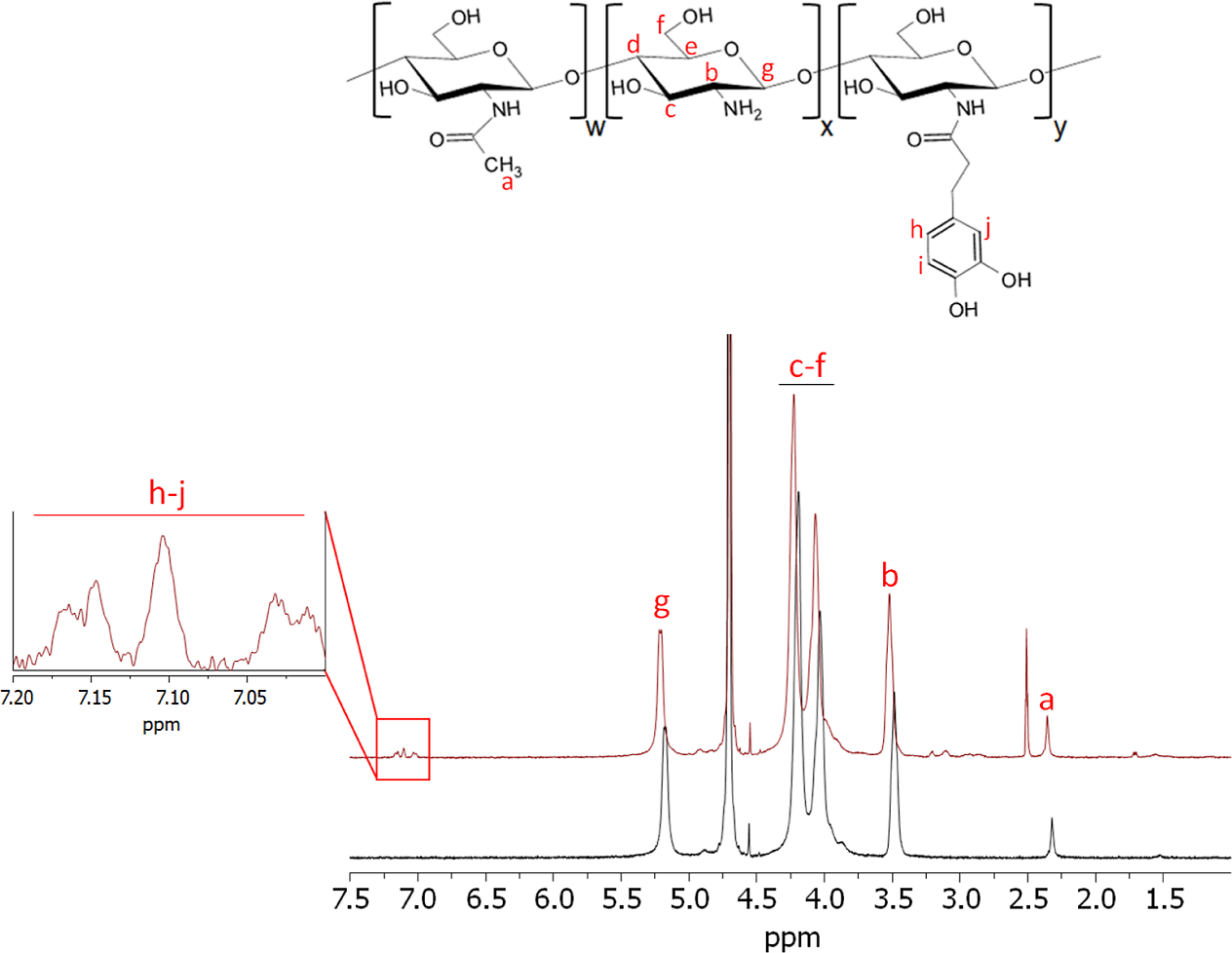
^1^H NMR spectra of Cs (black) and CsC (red) dissolved in a D_2_O/DCl mix (50:1 v/v) at 70 °C.

^1^HNMR spectra of Cs and CsC are shown in Figure 1. In the raw Cs spectrum, a distinct band can be observed at 2.32 ppm (a), which is attributed to the three *N*-acetyl protons. Additionally, a peak at 3.49 ppm (b) is observed, corresponding to the proton of the C-2 position.^52,53^ Furthermore, the multiplet observed between 4.03 and 4.20 ppm (c–f) is associated with the protons of the polysaccharide ring.^54^ The resonance band of the anomeric proton also appears at 5.18 ppm (g).^55^ The incorporation of catechol moieties in CsC was confirmed by observing a multiplet between 7.01 and 7.19 ppm, which is attributed to the aromatic protons (h–j).^56^

The DS(%) of catechol for CsC was calculated using UV spectrophotometry, yielding values of 2.98% ± 0.08%.

### Preparation and Characterization of Nanoparticulate PECs

Nanoparticulate PECs arise from robust electrostatic interactions between charged microdomains of at least two oppositely charged PEs. When solutions of polyanions and polycations are mixed, insoluble PECs form spontaneously under specific conditions. The formation of PECs is influenced by various factors, including the polymer chain rigidity, location of ionic sites, pH, precursor chemistries, temperature, mixing intensity, ionic strength, and other controllable parameters. While a few studies are reporting the production of NPs based on PECs containing Cs and ulvan, the synthesis typically involves the use of sodium tripolyphosphate (TPP) as a counterion.^57^ In contrast, this study presents the production of nanoparticulate complexes formed through direct interactions between Cs and ulvan. The physical-chemical characteristics of the resulting HA-uncoated and HA-coated NPs are outlined in Tables 1 and 2, respectively.

**Table 1:** Hydrodynamic Properties and ζ-potential of the HA-Uncoated NPs^a^^,^^b^^,^^c^^,^^d^^,^^e^

^×^_Cs/Ulvan_	macroscopic appearance	*D*_h_ (nm)	PDI (%)	ζ-potential (mV)
0.45	agg			
0.65	sus	296 ± 13	22.3 ± 1.3	+40.7 ± 0.1
0.85	sus	298 ± 3	25.1 ± 1.6	+47.7 ± 0.3
1.05	sus	420 ± 25	24.6 ± 0.6	+51.1 ± 0.5
1.25	sol			
^×^_CsC/Ulvan_	macroscopic appearance	*D*_h_ (nm)	PDI (%)	ζ-potential (mV)
0.45	agg			
0.65	agg			
0.85	Agg			
1.05	Sus	282 ± 3	27.3 ± 2.1	+48.5 ± 2.0
1.25	Sol			

a × _Cs/Ulvan_ = Cs/ulvan mass ratio.

b × _CsC/Ulvan_ = CsC/ulvan mass ratio.

c Sol = solution.

d Sus = suspension.

e Agg = aggregates.

**Table 2:** Hydrodynamic Properties and ζ-potential of the HA-Coated NPs^a^^,^^b^^,^^c^^,^^d^^,^^e^

×_Cs_–_U__NPs/HA_	macroscopic appearance	*D*_h_ (nm)	PDI (%)	ζ-potential (mV)
0.15	Sol			
0.35	Sus	274 ± 3	18.8 ± 1.6	–28.8 ± 0.2
0.75	Sus	194 ± 5	7.6 ± 0.6	–24.9 ± 0.2
1.15	Agg	187 ± 2	16.9 ± 1.0	–20.5 ± 0.3
×_CsC–U__NPs/HA_	macroscopic appearance	*D*_h_ (nm)	PDI (%)	ζ-potential (mV)
0.15	Sol			
0.35	Sus	181 ± 4	19.4 ± 0.2	–30.1 ± 0.6
0.75	Agg			
1.15	Agg			

a × _Cs–U NPs/HA_ = Cs–U NPs/HA volume ratio.

b × _CsC–U NPs/HA_ = CsC–U NPs/HA volume ratio.

c Sol = solution.

d Sus = suspension.

e Agg = aggregates.

The observation of opalescence serves as a macroscopic sign of NP production in aqueous suspensions. Table 1 lists the characteristics of five PECs of Cs or CsC with ulvan at varying mass ratios. The clarity in the dissolution of PECs with higher ratios indicates a lower concentration of NPs, whereas macroscopic aggregates are evident in samples with lower ratios. For the Cs–U NPs, opalescent suspensions were noted within a ratio range of 0.65 to 1.05. Specifically, PECs at a ratio of 1.05 exhibited a *D*_h_ exceeding 400 nm. In contrast, PECs at ratios of 0.65 and 0.85 displayed similar *D*_h_, approximately 300 nm, suggesting smaller sizes. NPs formulated at a mass ratio of 0.85 are deemed to be more efficient, requiring less ulvan for their production. Notably, aggregates were observed in the CsC–U NPs at mass ratios between 0.45 and 0.85, while no opalescence was detected in PECs at a mass ratio of 1.25. The narrower range for achieving opalescent suspensions, compared to Cs–U NPs, is attributed to the reduced availability of –NH_3_^+^ groups, as some are occupied by catechol groups. For all HA-uncoated NPs, PDI values were very similar. Positive ζ-potential was determined, consistent with previous findings for similar materials and its values indicated high degree of colloidal stability in all uncoated PECs, exceeding 30 mV.^58,59^ However, the magnitude of the ζ-potential decreased as the proportion of negatively charged ulvan increased. Based on these findings, Cs–U NPs and CsC–U NPs at mass ratios of 0.85 and 1.05, respectively, were selected for further research.

HA-coated NPs were produced by introducing preformed PECs of Cs or CsC with ulvan into a HA solution. The formation of these HA-coated NPs is primarily influenced by the positively charged amino groups on Cs or CsC, which interact with the negatively charged carboxyl groups of HA. Typically, the adsorption of polyanions onto the surface of positively charged NPs can result in aggregate formation, either due to electrostatic interactions or due to inadequate electrostatic stabilization.^60^ However, our HA-coated PECs display smaller *D*_h_ and PDI compared with those without HA coating. Although many studies suggest that coating generally results in an increase in particle size, there are some reports indicating that the opposite effect can also occur.^61,62^ The reduction in *D*_h_ and PDI is attributed to the multiple electrostatic interactions, which compact and stabilize the polymeric matrix at the same time. The characterization of the HA-coated NPs using different NPs/HA volume ratios is shown in Table 2. For the Cs–U/HA NPs produced with ratios of 0.75 and 1.15, similar NPs were obtained with *D*_h_ lower than 200 nm and significant reduction of the PDI compared to the uncoated PECs; however, it was possible to observe macroscopic aggregates in the sample of 1.15. Nevertheless, only CsC–U/HA NPs produced with a volume ratio of 0.35 showed opalescent characteristics due to the instability of CsC–U NPs, showing 181 nm of *D*_h_ and 19.4 nm of PDI. The coating with HA produced NPs with negative ζ-potential, indicating that the HA neutralizes –NH_3_^+^ groups of Cs and excess –COO^–^ groups are available on the surface. The magnitude of the ζ-potential depends on the NPs/HA ratio; the greater the amount of HA compared to the NPs, the greater thenegative ζ-potential. A similar effect has been previously observed with Cs-TPP NPs, where coating with HA either reduces the positive charge of Cs NPs or induces a shift toward a negative ζ-potential.^63,64^ Based on the results, subsequent studies will be carried out with Cs–U/HA NPs and CsC–U/HA NPs with a ratio of 0.75 and 0.35, respectively.

**Figure 2:**
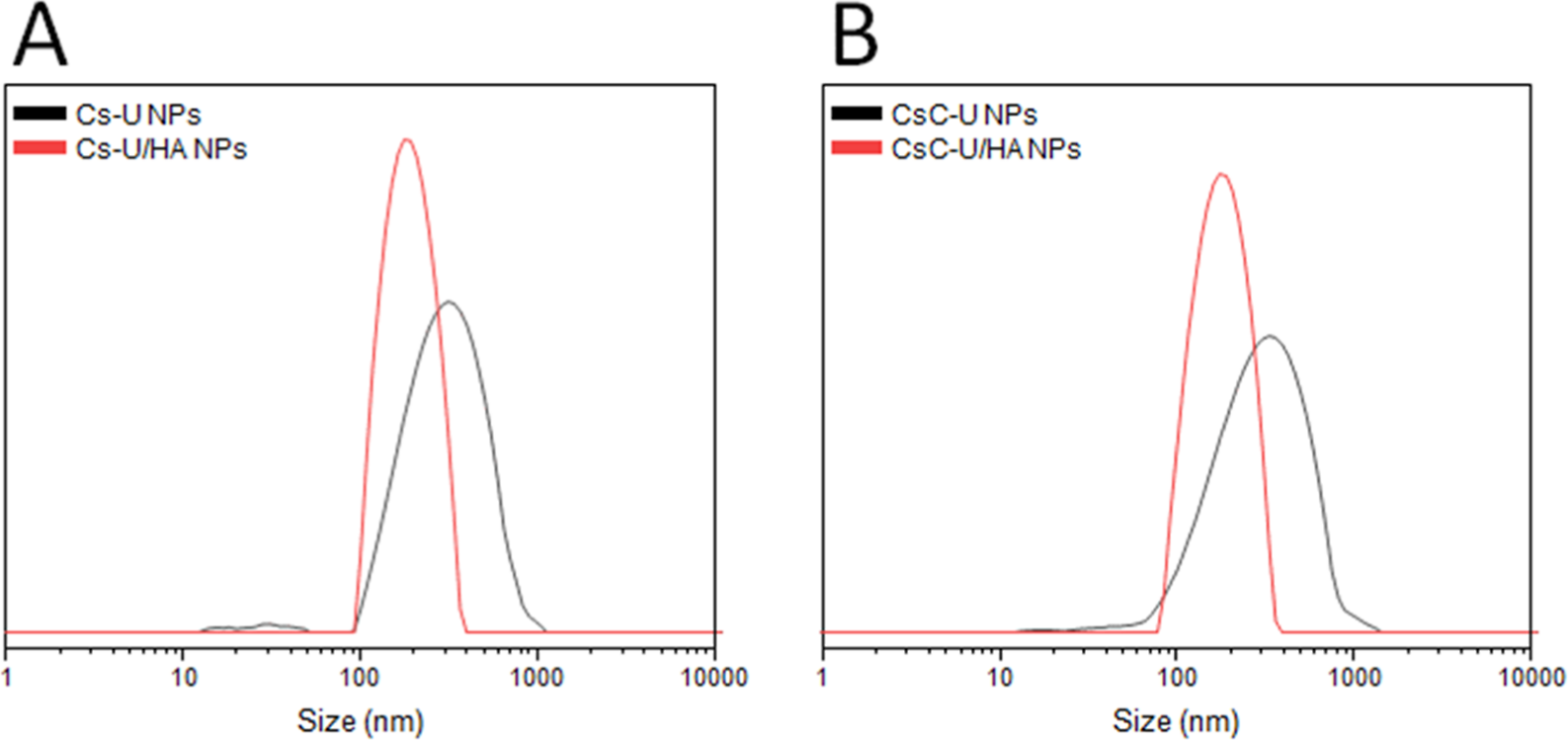
Size distribution of Cs–U NPs and Cs–U/HA NPs (A). Size distribution of CsC–U NPs and CsC–U/HA NPs (B).

The size distributions of both HA-coated and HA-uncoated NPs are depicted in Figure 2. Multiple prominent populations with varying *D*_h_ can be seen in the distribution of the Cs–U NPs, whereas the Cs–U/HA NPs exhibit unimodal distributions with a reduced PDI as shown in Figure 2A. The catecholic PECs demonstrated nearly identical behavior (Figure 2B). Our research group has noted that the HA coating had a comparable effect on NPs derived from Cs cross-linked with TPP.^50^

**Figure 3:**
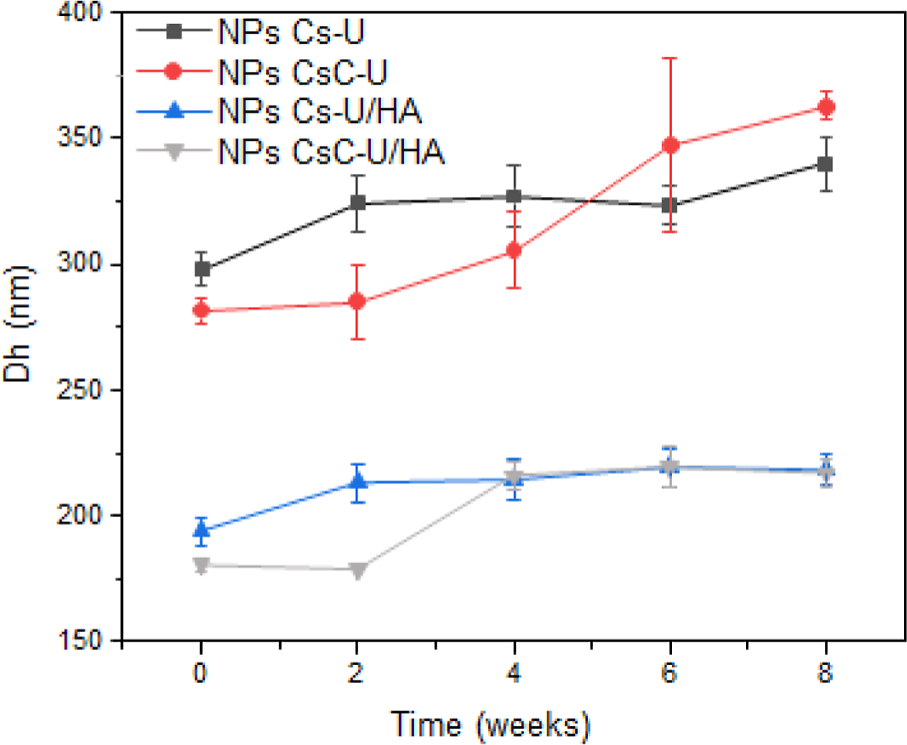
Comparison of *D*_h_ of the NPs, monitoring over time.

We continuously measured the *D*_h_ of Cs–U NPs, CsC–U NPs, Cs–U/HA NPs, and CsC/HA NPs for 8 weeks. As presented in Figure 3, the increase in *D*_h_ did not exceed 90 nm for the four NPs systems. The PECs with CsC showed a higher increasing *D*_h_ compared to those obtained with Cs. This behavior could be attributed to the orientation of the hydrophobic aromatic rings within the NPs, which may lead to the destabilization of the systems in an aqueous medium. Soliman et al. increased the stability of CsC-TPP NPs in different pH conditions through the oxidation of the catechol groups, using NaIO_4_ as an oxidizing agent. The oxidation process generates o-quinone groups, which can react covalently with the amino groups of the polysaccharide, forming a Schiff base.^65^ Additionally, coating the NPs with HA appears to enhance colloidal stability. This is evident from the observations made after an eight-week study, the *D*_h_ increased approximately 36 nm for the CsC–U/HA NPs, compared to the 81 nm observed for CsC–U NPs.

**Figure 4:**
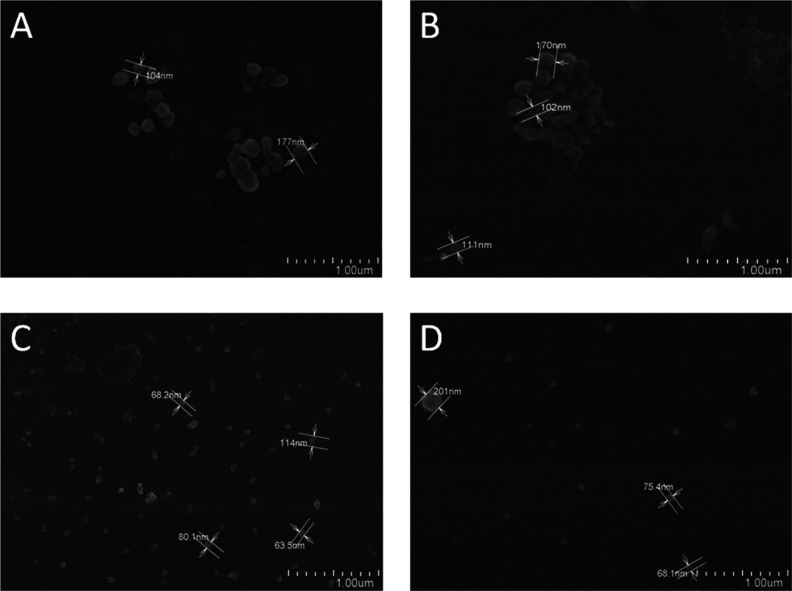
FE-SEM images of Cs–U NPs (A), CsC–U NPs (B), Cs–U/HA NPs (C), and CsC–U/HA NPs (D).

The morphology of the PECs was examined by using FE-SEM (Figure 4). All nanoparticulate materials exhibited a semispherical geometry with smooth surfaces. However, consistent with the DLS analysis, both Cs–U NPs and CsC–U NPs demonstrated larger sizes compared to the HA-coated NPs. The uncoated NPs exhibited sizes around 170 nm (Figure 4A,B), with a tendency toward the formation of aggregates of individual NPs. In contrast, images of Cs–U/HA NPs and CsC–U/HA NPs (Figure 4C,D) mostly depicted materials smaller than 100 nm with significantly lower polydispersity and greater separation between the materials. The FE-SEM results corroborate the effect of HA coating on size and PDI observed by DLS. However, notable discrepancies exist between the FE-SEM and DLS results, attributed to the collapse of the swollen state of the nanogels in a water medium, a consequence of the drying process involved in preparing samples for SEM analysis.^66^

**Figure 5:**
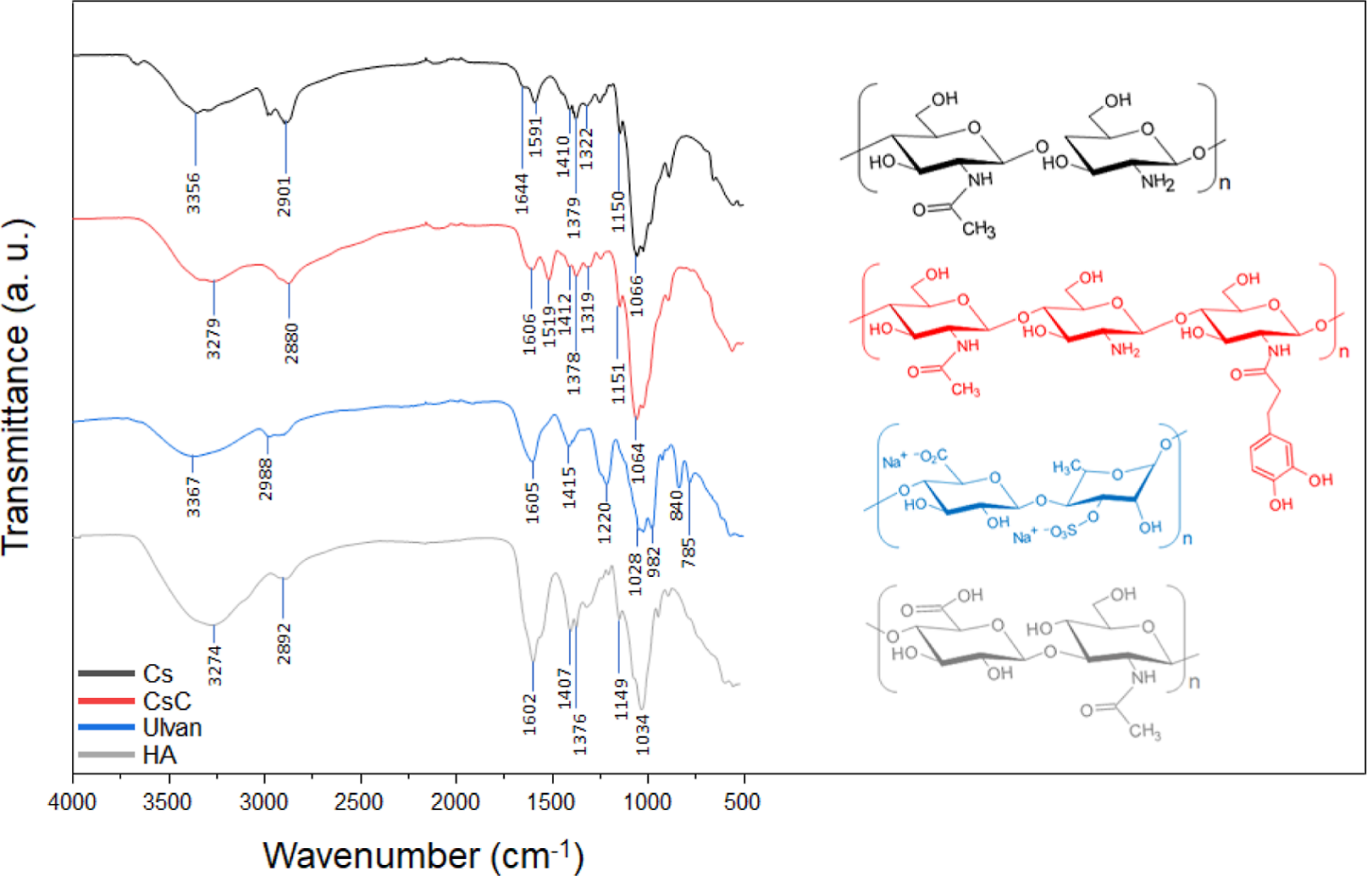
ATR-FTIR spectra of Cs, CsC, ulvan, and HA.

The results of the ATR-FTIR analysis of the polymers are listed in Figure 5. In the spectrum of Cs, characteristic bands at 3356, 2901 (O–H and N–H stretching vibrations, respectively), 1644 (amide I, due to the C=O stretching vibration for aliphatic primary amides), 1591 (amide II, due to the −N–H deformation vibration), 1410 (−CH_2_, bending), 1379 (−CH_3_, symmetrical deformation vibration), 1322 (amide III, due to C–N stretching vibration), 1150 (C–O–C, asymmetric strength vibration), and 1066 cm^–1^ (vibration of the pyranose structure) can be observed.^67^ In addition to the characteristic bands of Cs, the CsC spectra shows a peak at 1519 cm^–1^, attributed to the C–C aromatic stretching.^68^ The ulvan spectrum exhibits a symmetric stretching band at 1415 cm^–1^, attributed to the carboxylate groups. A peak corresponding to the stretching of C–O–S at 1220 cm^–1^ confirms the presence of sulfate ester substitution on rhamnoses. Furthermore, the bands at 840 and 785 cm^–1^ are not definitively indicative of axial O-2 position sulfate esters but are associated with the sugar cycles.^69^ The main peaks of the spectrum of HA at 1602, 1407, and 1034 cm^–1^ are attributed to C=O, COO–, and C–O–C stretching vibration, respectively.^70,71^

**Figure 6:**
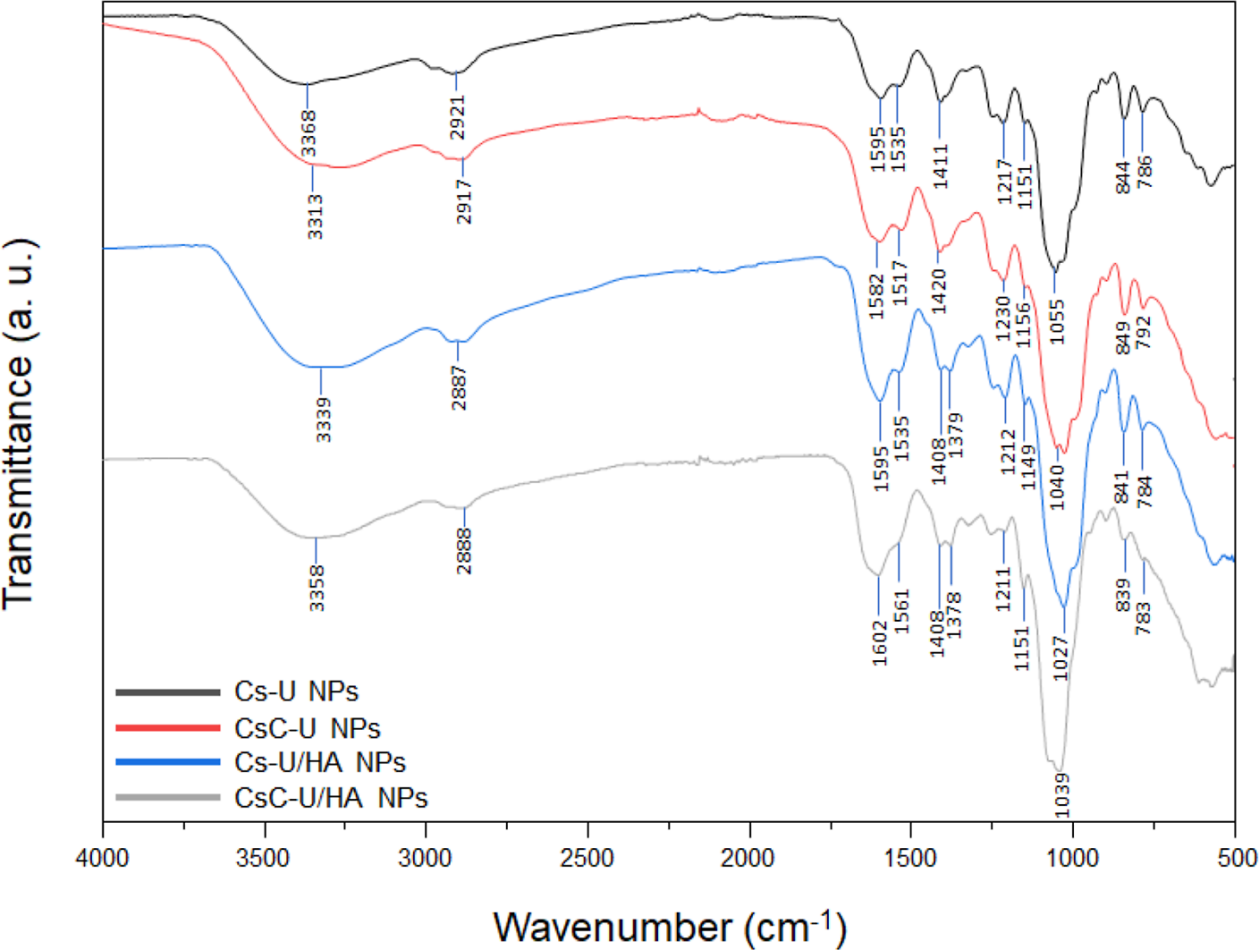
ATR-FTIR spectra of Cs–U NPs, CsC–U NPs, Cs–U/HA NPs, and CsC–U/HA NPs.

Figure 6 shows the spectra of the PECs. The bands of Cs at 3356, 2901, 1591, 1150, and 1066 cm^–1^ are shifted, indicating that such bonds are involved in a larger variety of chemical environments, including multiple hydrogen bonding that could be induced by the ionic cross-linking. On the other hand, the sulfate ester and sugar cycle bands of ulvan exhibited shifts in both the uncoated and HA-coated NPs, with values ranging from 1211 to 1230, 839 to 849, and 783 to 792 cm^–1^. Furthermore, in the HA-coated NP spectra, the peak attributed to C=O stretching vibration increased its intensity, and a shift in the peak corresponding to the carboxylate group of HA is exhibited, from 1407 to 1408 cm^–1^. These observations offer strong evidence of the role of the polysaccharides in the formation of the PECs.

### Antioxidant Activity of the NPs In Vitro

**Figure 7:**
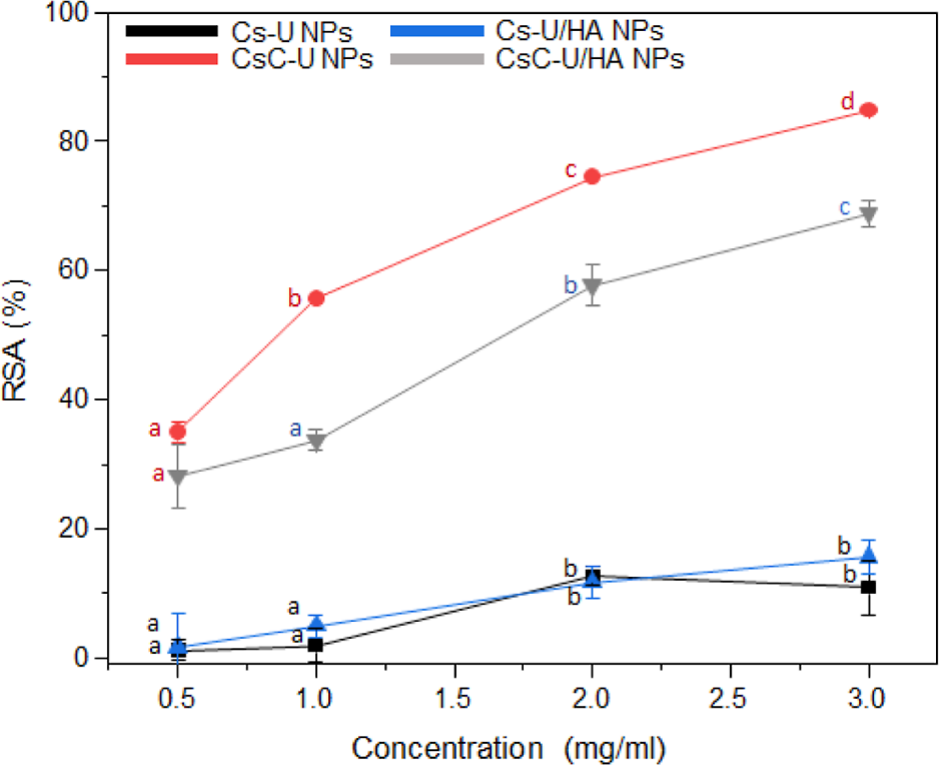
RSA of Cs–U NPs, CsC–U NPs, Cs–U/HA NPs, and CsC–U/HA NPs. Different types of NPs analyzed at the same concentration do not present significant differences if they have the same letter color. The same type of NPs analyzed at different concentrations do not present significant differences if they have the same letter.

The DPPH assay relies on measuring the scavenging activity of antioxidant compounds against free radicals. In this assay, the odd electron of the nitrogen atom in DPPH is reduced when it accepts a hydrogen atom from molecules possessing antioxidant capacity.^72^ In this study, the antioxidant activity, expressed as RSA, of the PECs was evaluated against varying concentrations (Figure 7). Both Cs–U and Cs–U/HA NPs exhibited less than 17% RSA at a concentration of 3.0 mg/mL. Conversely, the lowest concentration of CsC–U NPs demonstrated double the antioxidant behavior compared with the NPs lacking catechol incorporation. Furthermore, CsC–U NPs reduced more than 50% of the DPPH activity at a concentration of 1.0 mg/mL, and the most concentrated sample exhibited RSA close to 85%. These findings indicate that the incorporated HCA possesses hydroxyl groups capable of reacting with free radicals and neutralizing them, thus displaying excellent antioxidant potential.^73^ Previously, our research group demonstrated that CsC solutions with a concentration of 200 μg/mL exhibited a reduction in DPPH activity of over 85%.^50^ The reduction of the RSA shown by the NPs compared to the polymeric solutions is attributed to the multiple interactions between the polysaccharide chains that hide the catechol groups, limiting their availability and capacity to interact with free radicals. Additionally, the surface coating of the PECs with HA, as determined by ζ-potential, also reduces the availability of catechol groups, as the RSA of CsC–U/HA NPs decreases to 69% at a concentration of 3.0 mg/mL. However, the reduction in RSA of CsC–U/HA NPs compared to that of CsC–U NPs was only 16%, suggesting that much of their antioxidant activity is retained.

### Cytotoxicity Determination of the PECs

**Figure 8:**
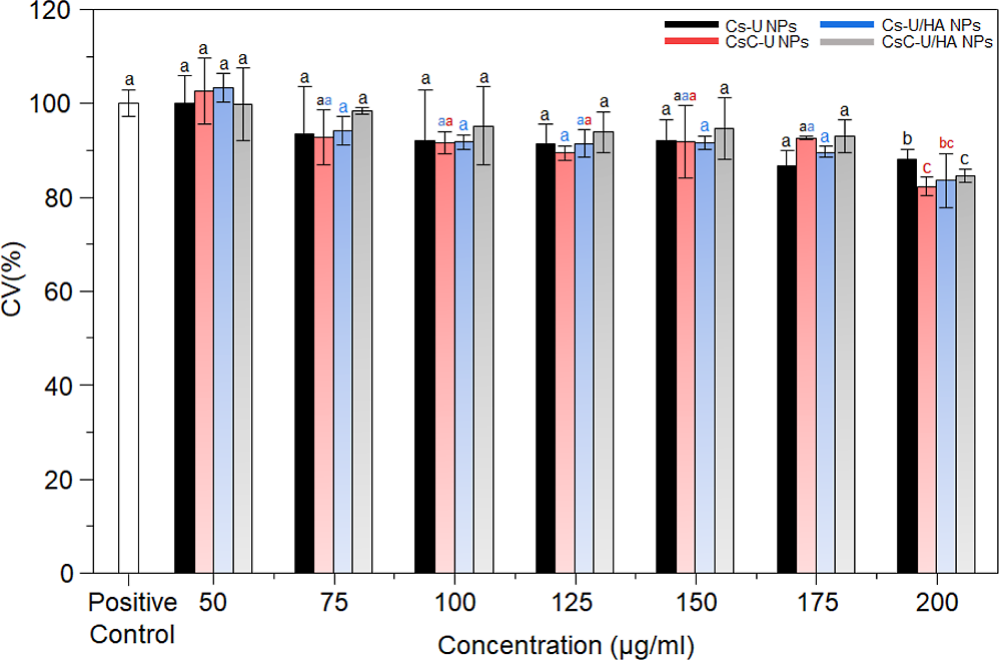
Cell viability on Arpe-19 exposed to the NPs. Different types of NPs analyzed at the same concentration do not present significant differences if they have the same letter. The same type of NPs analyzed at different concentrations do not present significant differences if they have the same letter color.

The biocompatibility of both HA-coated and uncoated NPs was evaluated in ARPE-19 cells using an MTT assay, which measures the activity of succinate dehydrogenase, a mitochondrial enzyme sensitive to toxic substances. For the cytotoxicity assessment, cells were exposed to various concentrations of Cs–U NPs, CsC–U NPs, Cs–U/HA NPs, and CsC–U/HA NPs. According to the results shown in Figure 8, all of the tested NPs exhibited minimal cytotoxicity at all administered doses. Specifically, Cs–U NPs had no significant impact on cell viability (*P* = 0.4), whereas Cs–U/HA NPs demonstrated a dose-dependent reduction in cell viability (*P* = 0.007). Conversely, CsC–U NPs displayed an interesting behavior: enhancing cell proliferation at lower concentrations but showing no significant differences at the highest doses (*P* = 0.06). For CsC–U/HA NPs, there were no cytotoxic effects observed at any of the tested concentrations (*P* = 0.1). The CV(%) was approximately 100% at the lowest concentration, with a decrease of less than 20% observed at 200 μg/mL. In addition, a significantly lower cytotoxicity of Cs–U NPs compared to the catecholic PECs can be observed at a concentration of 200 μg/mL. However, at all concentrations, the CV(%) remained above the threshold set by the ISO standard for biocompatibility.^74^ This aligns with findings from da Silva et al., who reported similar results for Cs NPs encapsulating rosmarinic acid tested in ARPE-19 cell cultures at concentrations below 1 mg/mL.^75^

## Conclusion

In this study, we report for the first time the synthesis and characterization of nanoparticulate PECs based on Cs, CsC, ulvan, and HA. The functionalization of Cs with catechol groups was achieved, as confirmed by ^1^H NMR and UV spectrophotometry. Polymeric NPs were obtained in a water suspension due to electrostatic interactions between the chitosans and ulvan. The coating of the NPs with HA was evidenced by the attainment of materials with a negative surface charge, resulting in good stability and a reduction in their *D*_h_ and polydispersity compared to the uncoated ones. Additionally, FE-SEM analysis confirmed the impact of the HA coating on the size of the NPs. The presence of the PEs in the nanoparticulate materials was confirmed by ATR-FTIR results. PECs containing CsC exhibited a significant enhancement in the RSA against DPPH radicals compared to the other Cs-based materials, with the HA coating only reducing the activity by 16%. Studies conducted in cell cultures have shown that the PECs were nontoxic at the concentrations tested. The nanomaterials obtained from these PECs hold promise as potential drug delivery systems for various biotechnological and biomedical applications. This includes the microencapsulation of drugs, enzymes, cells, and microorganisms as well as the creation of complexes with polynucleotides or oligonucleotides for use as vectors in gene therapy.

## Supporting Information Available

Calibration graph of HCA solutions at different concentrations (PDF)

## Abbreviations

^1^H NMR: ^1^H nuclear magnetic resonance
ATR-FTIR: attenuated total reflection-Fourier transform infrared
Cs: chitosan
CsC: chitosan–catechol
Cs–U NPs: nanoparticles of Cs–ulvan
CsC–U NPs: nanoparticles of CsC–ulvan
Cs–U/HA NPs: nanoparticles of Cs-ulvan/hyaluronic acid
CsC–U/HA NPs: nanoparticles of CsC-ulvan/hyaluronic acid
CV(%): percentage of cell viability
*D*_h_: hydrodynamic diameter
DLS: dynamic light scattering
DPPH: 2,2-diphenyl-1-picrylhydrazyl
DS(%): degree of substitution
DW: deionized water
EDC: *N*-(3-(dimethylamino)propyl)-*N′*-ethylcarbodiimide hydrochloride
FE-SEM: field emission-scanning electron microscopy
HA: hyaluronic acid
HCA: hydrocaffeic acid
MTT: 3-(4,5-dimethylthiazol-2-yl)-2,5-diphenyl-tetrazolium bromide
NPs: nanoparticles
PDI: polydispersity index
PECs: polyelectrolyte complexes
RSA: radical scavenging activity
TPP: sodium tripolyphosphate
