# Supplementary material for: Production and Characterization of Nanoparticulate Polyelectrolyte Complexes of Chitosan–Catechol, Ulvan, and Hyaluronic Acid
Source: ACS Omega. 2025 Jan 17;10(4):3474–85. doi: 10.1021/acsomega.4c07429 (PMC11800033; doi:10.1021/acsomega.4c07429)
Supplement: Supplementary file 1 — ao4c07429_si_001.pdf [file ao4c07429_si_001.pdf]

Production and characterization of nanoparticulate polyelectrolyte complexes of chitosan−catechol, ulvan and hyaluronic acid

Francisco J. Caro-León[a,b,], Erika Silva-Campa[b], René A. Navarro-López[b], Daniel Fernández-Quiroz[c], Vivian A. Figueroa-León[d], Maria A. Trujillo-Ramirez[d], Luis Miguel López-Martínez[e,f], Maria Rosa Aguilar[a,g], Osiris Álvarez-Bajo[b,h]*

[a] Instituto de Ciencia y Tecnología de Polímeros (ICTP), CSIC, 28006 Madrid, Spain

[b] Departamento de Investigación en Física de la Universidad de Sonora (DIFUS), 83000 Hermosillo, México

[c] Departamento de Ingeniería Química y Metalurgia, Universidad de Sonora, 83000 Hermosillo, México

[d] Programa de Ingeniería Biomédica, Universidad de Sonora, 83000 Hermosillo Sonora

[e] Departamento de Investigación en Polímeros y Materiales (DIPM), Universidad de Sonora, 83000 Hermosillo Sonora

[f] Universidad Estatal de Sonora (UES), Av. Ley Federal del Trabajo s/n, Col. Apolo, 83100 Hermosillo, Sonora

[g] CIBER de Bioingeniería, Biomateriales y Nanomedicina, Instituto de Salud Carlos III, 28029 Madrid, Spain

[h] Consejo Nacional de Humanidades Ciencia y Tecnología CONAHCyT, Ave. Insurgentes Sur 1582, Col. Crédito Constructor, Benito Juárez, 03940, CDMX, Mexico

Supporting Information

Production and characterization of nanoparticulate polyelectrolyte complexes of chitosan−catechol, ulvan and hyaluronic acid

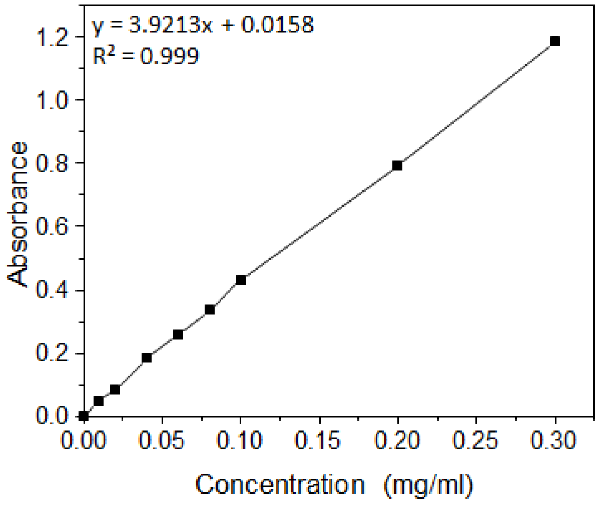

Figure S1. Calibration graph of HCA solutions at different concentration (λ = 280 nm).
